# The indirect effect of compassion on katagelasticism: the mediatiang role of moral disengagement and the moderating effect of intolerance of uncertainty

**DOI:** 10.1186/s40359-023-01063-z

**Published:** 2023-01-26

**Authors:** Alexandra Maftei

**Affiliations:** Faculty of Psychology and Education Sciences, Alexandru Ioan Cuza University, Iasi, Romania

**Keywords:** Katagelasticism, Moral disengagement, Intolerance of uncertainty, Compassion

## Abstract

The junction between moral psychology, humor, and some specific personality variables (i.e., uncertainty intolerance and compassion toward others) has been a neglected field of study. The present research explored the role of moral disengagement and intolerance of uncertainty in the relationship between compassion and katagelasticism. The sample was formed by 763 adults aged 18 to 70 (*M* = 24.62, *SD* = 8.29, 73.9% women). The findings suggested significant negative associations between compassion and moral disengagement and positive correlations between katagelasticism, moral disengagement, and intolerance of uncertainty. Furthermore, moral disengagement mediated the link between compassion and katagelasticism, while intolerance of uncertainty moderated the link between compassion and moral disengagement. Significant gender differences were also suggested concerning all our study's primary variables, with women scoring higher in the compassion and intolerance of uncertainty and significantly lower than men in the moral disengagement and katagelasticism dimensions. The results are discussed regarding their theoretical and practical implications related to moral disengagement and the underlying personal factors.

## Introduction

Humor is a socially desirable trait [1]. The social function of humor in interpersonal relationships has been explored in various studies highlighting its many benefits for the individual and their interpersonal relationships [2]. However, when discussing humor, we also acknowledge its dark side, which leads to its socially malevolent expressions [3]. More specifically, we can discuss humor from a bright point of view, i.e., benevolent or moral humor [4], as well as from a darker side, i.e., malevolent humor which can generate various negative reactions [5].

In the current research, I was interested in *katagelasticism*, a subclinical individual factor related to the personality pathology trait of antagonism [6] that describes people who enjoy exploiting others’ mistakes without caring about their subsequent reactions but rather satisfying their desire to laugh at them [5]. According to Martin and Ford [6], “katagelasticists actively seek out situations where they can make fun of others and laugh at their foibles, mishaps, or defects” and “show little concern for the feelings of others whom they put down or laugh at” (p. 136). Furthermore, the authors continue, katagelasticism do not feel responsible for making others’ suffer when they laugh at them, since they consider laughter as inherently positive.

## Literature review

The role and consequences of katagelasticism in social situations have been explored in studies that generally suggest its potentially adverse outcomes. For example, katagelasticism was suggested as a significant predictor of bullying perpetration across age groups [7–9] and online trolling [10]. Also, katagelasticism seems to predict conflict, disagreement, and jealous behaviors in romantic relationships [11, 12]. Thus, it is all the more important to explore katagelasticism and its junction with compassion toward others, uncertainty intolerance, and moral disengagement, especially since there is scarce evidence available in this regard.

Previous research suggested that individuals high in katagelasticism are usually self-centered and generally unfriendly, less happy [4, 13] and more prone to vulnerable narcissism [14]. Katagelasticism was also found to be significantly related to psychopathic personality traits, such as Machiavellianism, psychopathy, and a generally manipulative and impulsive lifestyle and callousness [9, 10]. Furthermore, individuals high in katagelasticism are generally described by extraversion and low agreeableness and conscientiousness [15]. Similar to katagelasticism, *schadenfreude* describes an expression of an aggressive humor style reflected in one’s tendency to gain pleasure or satisfaction from others’ misery [16] due to internal predispositions [17] such as those related to social aversiveness or insecurity [18]. Furthermore, other previous studies considered schadenfreude as one facet of one’s lack of morality [19]. Though these findings are not explicitly related to katagelasticism, one of the primary variables in the current study, it is essential to acknowledge the similarities and almost overlapping specificities of the two concepts and their link to morality and uncertainty to understand katagelasticism better.

### Humor and morality

Previous research investigating humor and morality focused on light and dark humor and their link with moral violations [20]. Other investigations suggested that individuals with internalized moral identities are perceived as less humorous [21]. McGraw and Warren [22] investigated the conditions that make a violation benign (from a humorous point of view) and found that one of them was related to the psychological distance from the violation, which can also be translated as a form of moral disengagement. However, though previous research investigated several facets of humor and moral disengagement (e.g., disparagement humor, a specific type of denigrative humor; [23, 24], the link between the joy felt when laughing at other people (i.e., katagelasticism) and the cognitive mechanisms that allow people to morally disengage from unethical behavior have not yet been addressed in previous studies. Thus, the present study aimed to fill in this gap by extending the related knowledge and adding findings related to the potential role of moral disengagement when investigating people’s propensity to use laugh at others.

Furthermore, previous research investigated disparagement humor and moral disengagement [24]. However, the link between the joy felt when laughing at other people and the cognitive mechanisms that allow people to disengage from unethical behavior morally seem to lack sufficient evidence. Thus, the present study investigated the relationships between compassion and katagelasticism through moral disengagement.

### Moral disengagement, intolerance of uncertainty, and compassion

The social cognitive theory of moral agency [25–27] describes moral disengagement as a cognitive self-regulation mechanism that facilitates people's engagement in immoral behavior without apparent remorse or guilt. In other words, when moral disengagement mechanisms are activated, they decrease the intensity of personal arousal in situations involving high personal costs [28]. According to Bandura's theory, moral disengagement serves as a disinhibitory mechanism, allowing individuals to behave immorally due to eight primary psychological means placed into four categories, i.e., (1) the cognitive restructuring of the immoral, harmful behavior, (2) disregarding or distorting the consequences of harmful behavior, (3) blaming and dehumanizing the victim, as well as (4) minimizing or disregarding one's role in causing harm [25–27].

The cognitive restructuring of the immoral, harmful behavior implies *moral justification*, *euphemistic labeling*, and *advantageous comparison*, all of these comprising the arguments and beliefs that frame immoral behaviors in a positive light. For example, characterizing a harmful behavior as serving a moral purpose (i.e., "It’s for the greater good”) indicates the moral justification mechanism of moral disengagement. *Disregarding or distorting the consequences* of harmful behavior describes strategies that allow people to distance themselves from the harmful outcomes of their behavior or highlight the positive rather than negative consequences associated with their immoral behavior. Furthermore, *blaming and dehumanizing the victim* refers to how individuals consider the victim responsible for the harm they receive (e.g., “They deserved it!/ They brought it to themselves”). Finally, *minimizing or disregarding one’s role in causing harm* refers to the cognitive strategies that contribute to the diffusion or displacement of responsibility for one’s harmful acts by minimizing or disconsidering one’s contribution and responsibility, and rather attributing it to a higher group authority (e.g., “Everybody is doing it", or “We made that decision together, I wasn’t the only one who decided”) [29].

There are various models describing the development of moral disengagement and its consequences. For example, in their additive model for the development of moral disengagement, Hyde and their collaborators [30] described factors related to the home environment, neighborhood impoverishment, as well as personal variables such as empathy as the primary variables shaping the developmental trajectory of moral disengagement, further leading to antisocial behavior. A more recent review by Newman and their collaborators [31] indicated several factors at both teams and organizational levels and personal characteristics such as low empathy, male gender, and cynicism that would further lead to various deviant behaviors. Regardless of the working model, the related research highlights the various adverse effects of moral disengagement, e.g., aggression, bullying, cyber aggression, and antisocial behavior, in general [32–35]. Thus, extending and adding to the previous findings related to the predictors and mediators of moral disengagement is all the more important.

People’s tendencies to morally disengage have been explored concerning various personal factors. Previous studies highlighted the important link between moral disengagement and intolerance of uncertainty (i.e., “the excessive tendency of an individual to consider unacceptable that a negative event may occur, however, small the probability of its occurrence”; [31], p. 552) in various contexts. For example, Maftei and Holman [36] suggested a significant positive link between intolerance of uncertainty and moral disengagement in the COVID-19 pandemic. Other researchers identified a similar link in organizational contexts [37]. However, in the context of humor, especially the dark side of humor, the relationship between the two concepts (i.e., moral disengagement and intolerance of uncertainty) remains unclear.

We also know from previous studies that moral disengagement can be predicted by low levels of empathy, moral identity, and reflective moral attentiveness [38], which is generally positively associated with Machiavellianism, cynicism, external locus of control, and moral relativism, and positively linked with moral identity, moral idealism, empathetic concern, guilt, honesty-humility, conscientiousness, and agreeableness [39–42]. Furthermore, out of the eight mechanisms of moral disengagement [27], “dehumanization is a key mechanism that operates by nullifying self-restraints operating through feelings of empathy and compassion” ([43], p. 374). Though the relationship between compassion (towards others) and moral disengagement remains a topic that needs further research, research usually points to their generally negative association [44].

### Katagelasticism, intolerance of uncertainty, and compassion

Though several studies explored the links between katagelasticism and various personality traits, there is still a need for further findings related to the roles of intolerance of uncertainty and compassion. For example, the Intolerance of Uncertainty Model of Generalized Anxiety explored by Kuiper and their collaborators [45] suggested that people’s higher intolerance of uncertainty might decrease adaptive humor. Furthermore, the authors highlighted that increased levels of stress and intolerance of uncertainty seem to suppress self-enhancing humor, favoring maladaptive humor-related coping strategies, e.g., self-defeating humor.

Previous research highlighted the significant link between katagelasticism and various dark traits, such as Machiavellianism, psychopathy, and callousness [7, 10, 46, 47]. Similarly, other studies pointed to the links between katagelasticism, low agreeableness, and low conscientiousness [15]. Furthermore, [48] suggested a potential link between katagelasticism and emotional intersocial sensitivity or compassion. More specifically, the authors suggested that, as previously shown in less recent investigations [49], aggressive personality traits are related to the enjoyment of aggressive, humorous stimuli, while empathy might have a contrasting role by contributing to less enjoyment of disparagement humor [50]. However, regarding compassion and katagelasticism, the available data is scarce, and the present study aimed to fill this gap by also addressing the future research direction proposed by [48].

## The present study

Though previous research has looked into several aspects of humor and moral disengagement [23, 24], to our knowledge, no previous research has explored the link between compassion, the joy felt when laughing at other people, and the potential mediating roles of moral disengagement. Also, the knowledge related to the potential moderating role of intolerance of uncertainty concerning the link between compassion and moral disengagement is scarce, especially when investigating the further links to katagelasticism.

The detrimental effects of moral disengagement have been assessed in various studies, all of them highlighting the importance of further exploring the potential predictors and associated factors of these cognitive mechanisms [32–35]. However, the specific indirect effect of moral disengagement on katagelasticism has not yet been investigated when discussing compassion. For example, though some previous data suggested a significant relationship between disparagement humor and moral disengagement, the specific link with katagelasticism still needs further investigation. Similarly, the relationship between humor and morality [20, 22–24], as well as between katagelasticism and different facets of compassion [45, 48, 50] need further investigation. As a result, the current study aimed to address these gaps by expanding the existing knowledge and adding data about the potential mediating role of moral disengagement on the link between compassion and katagelasticism and the moderating role of intolerance of uncertainty.

Based on the previous findings related to the links between the variables of interest, the present’s study primary assumptions were: *H1.* Compassion toward others would be negatively linked moral disengagement and katagelasticism [44]; *H2*. Intolerance of uncertainty would be positively linked to moral disengagement and katagelasticism [36, 37, 51]; *H3.* Intolerance of uncertainty would moderate the link between compassion toward others and moral disengagement [45]; *H4.* Moral disengagement would mediate the link between compassion and katagelasticism [19, 48].

## Method

### Participants and procedure

The current sample was formed by 763 adults aged 18 to 70 (*M* = 24.62, *Mdn* = 21, *SD* = 8.29, 73.9% women). I used the convenience sampling approach [52]. All participation was voluntary. Most participants were students enrolled at the university where the author is affiliated; in their case, the participation was rewarded with course credits. For non-students, no rewards were offered. The participation link was also distributed in students’ social media groups (university-related, e.g., online groups created by specific faculties to share research materials, study books, or general ideas about their academic syllabus) and other online communication platforms (e.g., Whatsapp). All participants were informed that there were no right or wrong answers and that they could leave the study at any time. Furthermore, all participants were informed about the anonymity and confidentiality of their answers and that they could retire from the study at any time, including after their beginning to answer the study’s questions. The only inclusion criteria were related to age (> 18).

The participants filled in measures assessing moral disengagement, intolerance of uncertainty, katagelasticism, compassion, and a demographic scale. The average time needed to answer all the questions was around 20 min. The study protocol was designed according to the ethical requirements of the Ethics Committee, where the author is affiliated, following the Declaration of Helsinki and the national laws from Romania regarding ethical conduct in scientific research, technological development, and innovation.

### Measures

**Katagelasticism** The ten items measuring katagelasticism from the short form of the scale developed by Ruch and Proyer (PhoPhiKat-30; [53]) were further used. The items measure a general score using a Likert scale ranging from 1 (strongly disagree) to 4 (strongly agree). Example items include statements such as *When related to making jokes or funny remarks about other people I rather follow the motto *“*An eye for an eye, a tooth for a tooth*”* than *“*If someone strikes you on the right cheek, offer him the other also*”, and “*If other people poke fun at me than I pay them back in the same way— but more so*”. Higher scores indicated higher katagelasticism.

**Compassion** The Compassion Scale [54] assessed the total compassion score for the current sample of participants. The scale consists of 16 items measured on a 5-point Likert scale, ranging from 1 (almost never) to 5 (almost always). Example items include statements such as “I pay careful attention when other people talk to me about their troubles” and “If I see someone going through a difficult time, I try to be caring toward that person”. Higher scores indicated higher levels of compassion.

**Moral disengagement** The Moral Disengagement Scale developed by Moore and their collaborators [55] was used, a measure developed from Bandura’s moral disengagement framework [25–27]. The instrument consists of 8 items measuring each of the eight moral disengagement mechanisms, i.e., Moral Justification (i.e., It is okay to spread rumors to defend those you care about), Euphemistic Labelling (i.e., Taking something without the owner’s permission is okay as long as you’re just borrowing it), Advantageous Comparison (i.e., Considering the ways people grossly misrepresent themselves, it’s hardly a sin to inflate your own credentials a bit), Displacement of Responsibility (i.e., People shouldn’t be held accountable for doing questionable things when they were just doing what an authority figure told them to do), Diffusion of Responsibility (i.e., People can’t be blamed for doing things that are technically wrong when all their friends are doing it too), Distortion of Consequences (i.e., Taking personal credit for ideas that were not your own is no big deal), Dehumanization (i.e., Some people have to be treated roughly because they lack feelings that can be hurt), Attribution of Blame (i.e., People who get mistreated have usually done something to bring it on themselves). Participants answered on a 7-point Likert scale ranging from strongly disagree (1) to strongly agree (7). I used the scale's total score, and higher scores reflected higher moral disengagement.

**Intolerance of uncertainty** was measured using the Intolerance of Uncertainty Scale-Short form (IUS-S), developed by [56]. The scale comprises 12 items which participants answered on a 5-point Likert scale, ranging from 1 (not at all characteristic of me) to 5 (Entirely characteristic of me), with high scores indicating high intolerance of uncertainty. The items measure a general score, and example items include statements such as *Unforeseen events upset me greatly*, and *The smallest doubt can stop me from acting*.

The back-translation method was used to translate the scales from English into the Romanian language [57]. A demographic scale assessed participants’ gender and age.

### Overview of statistical analysis

Data cleaning steps were performed preliminary to the analyses. Next, I computed the Skewness and Kurtosis values for the primary variables (i.e., katagelasticism, intolerance of uncertainty, moral disengagement, and compassion) to assess the normality of the distributions. The values were in the [− 1; 1] range; thus, the data were normally distributed, and I further used parametric tests [58, 59].

Next, I performed univariate analysis (i.e., descriptive statistics). A preliminary analysis was conducted to examine how the demographic variables (i.e., age, gender) were related to participants’ moral disengagement using the IBM SPSS 26v. program. I further performed zero-order correlations assessed the associations between the study’s primary variables. Then, I performed mediation analyses using the Hayes [60] SPSS macro program PROCESS. I used Model 7 to explore the moderating effects of intolerance of uncertainty on the link between compassion and moral disengagement and the mediating effect of moral disengagement on the relationship between compassion and katagelasticism. For indirect effects, 95% confidence intervals (CI) with 5000 bootstrapped samples were computed and tested regarding statistical significance.

## Results

### Preliminary analyses

Descriptive statistics for the main study variables are presented in Table 1. Preliminary analyses indicated that participants’ age was not significantly associated with moral disengagement, katagelasticism, or compassion. However, results suggested that age was significantly and negatively associated with participants’ intolerance of uncertainty. More specifically, the younger the participants, the higher the levels of intolerance to uncertainty (*r* = 0.07, *p* = 0.04). No other significant associations were found between participants’ age and the study’s main variables.

**Table 1:** Descriptive statistics, T-test results (gender), and zero-order correlations among the primary variables (N = 766)

	M	SD	Min	Max	α	t-test	Hedge’s g	1	2	3	4
1. Moral disengagement	21.63	10.00	8	56	.86	6.01**	.51	–			
2. Katagelasticism	19.53	6.31	10	40	.86	6.68**	.57	.72**	–		
3. Intolerance of uncertainty	31.96	9.82	12	60	.90	− 3.75**	.30	.16**	.14**	–	
4. Compassion	62.11	9.57	21	80	.86	− 5.81**	.49	− .41**	− .37**	.02	–
5. Age	24.62	8.29	18	70	–	–	–	− .05	− .04	− .07*	− .06

*p < .05; **p < .001; α = Cronbach’s alpha for internal consistency

Results also suggested significant gender differences concerning all the primary variables (all *p*-s < 0.001). T-test results indicated that men scored significantly higher than women concerning moral disengagement (*M*_men_ = 25.34, *M*_women_ = 20.33), katagelasticism (*M*_men_ = 22.13, *M*_women_ = 18.61), and significantly lower on the intolerance of uncertainty (*M*_men_ = 29.76, *M*_women_ = 32.71) and compassion *(M*_men_ = 58.72, *M*_women_ = 63.31) dimensions. Results from the zero-order correlations among the primary variables indicated that moral disengagement was significantly and positively associated with katagelasticism (*r* = 0.72, *p* < 0.001) and intolerance of uncertainty (*r* = 0.16, *p* < 0.001) and negatively with compassion (*r* = − 0.47, *p* < 0.001).

### Testing For moderated mediation

The hypothesized moderated mediation model was tested in a single model (Model 7) using a bootstrapping approach to assess the indirect effects of moral disengagement on the link between compassion and katagelasticism at differing levels of the moderator, i.e., intolerance of uncertainty. I chose this model because it explicitly tests the moderating effect on the predictor-to-mediator path. I used the index of moderated mediation to assess the significance of the assumed differences in the indirect effects across levels of intolerance uncertainty. Significant effects were indicated by the absence of zero within the confidence intervals.

**Fig. 1:**
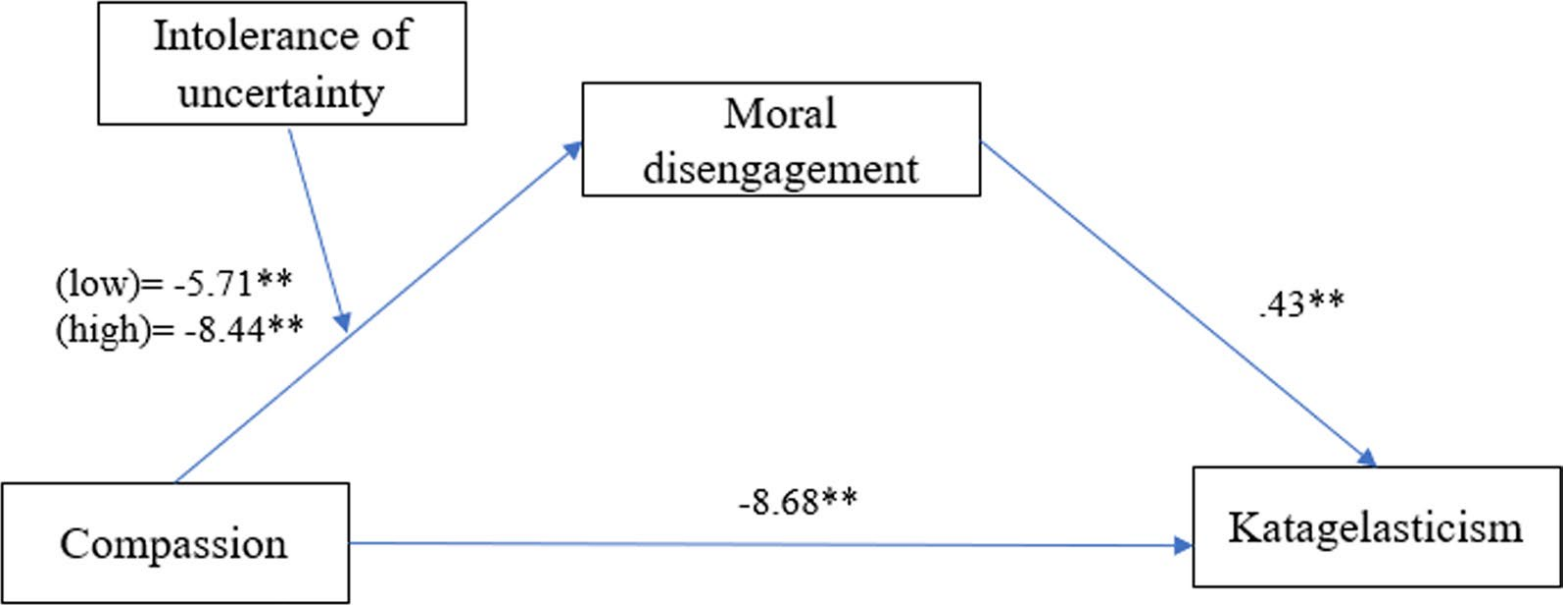
The moderated effect of moral disengagement on the link between compassion and katagelasticism. Values represent unstandardized coefficients. ** *p* < .001

The results indicated that intolerance of uncertainty significantly moderated the link between compassion and moral disengagement (*b* =− 0.13, *SE* = 0.06, *p* = 0.04, 95% CI [− 0.27; − 0.004], *R*^2^chng = 0.007. The conditional effects of the predictor at low (− 1 *SD*), medium (mean), and high (+ 1 *SD*) levels of the moderator were all significant (all *p*-s < 0.001). The direct effect of compassion on katagelasticism was significant, *b* =− 0.08, *SE* = 0.03, *p* = 0.008, 95% CI [− 1.50; − 0.22]. Moral disengagement significantly predicted katagelasticism, *b* = 0.43, *SE* = 0.01, *p* < 0.001, 95% CI [0.40; 0.47], *R*^2^ = 0.52 and had a significant negative indirect effect on the link between compassion and katagelasticism, *b* =− 0.06, *SE* = 0.03, *p* = 0.008, 95% CI [− 1.50; − 0.22]. The overall moderated mediation model was supported with the index of moderated mediation =− 0.06 (95% CI = − 0.11; − 0.005). The conditional indirect effect was strongest in those high in intolerance of uncertainty (1 SD above the mean; effect =− 8.44, *SE* = 0.97, 95% CI = − 10.36; − 6.53) and weakest in those with low levels of intolerance of uncertainty (1 SD below the mean, effect = − 5.71, *SE* = 0.82, 95% CI = − 7.34; − 4.80) (see Fig. 1.). The pattern of results was similar when the model was ran using gender as a covariate.

## Discussion

Overall, the present results suggested a significant indirect effect of compassion on katagelasticism through moral disengagement, moderated by intolerance of uncertainty. I found that participants low in compassion and high on intolerance of uncertainty are more likely to morally disengage and to enjoy laughing at other people (i.e., use katagelasticism). One of the primary findings from the current research suggested that moral disengagement was significantly linked to katagelasticism. This means that individuals who usually engage in cognitive mechanisms that would subsequently ease their moral discomfort generated by unethical behaviors are also more prone to take pleasure in laughing at others.

Though in need of further research using large and more heterogeneous samples, the present results underlined the complex ways the joy of laughing at others is related to the cognitive restructuring of immoral behaviors. Cognitive mechanisms underlying moral disengagement, such as moral justification, blaming the victim, euphemistic labeling, dehumanization, or advantageous comparison, seem to frame unethical conduct using hostile humor indirectly through (low) compassion and (high) intolerance of uncertainty. The joy of laughing at others might lead to cognitions that minimize the consequences (“*So, what if I am laughing? They deserve it!*”, i.e., dehumanization) through low levels of compassion towards the targeted person or low levels of tolerance towards uncertainty. Though the previous related literature did not specifically address this mediation model, the present results align with other investigations highlighting the significant, positive relationship between (high) intolerance of uncertainty, katagelasticism, and moral disengagement.

McGraw and Warren [22] suggested that the psychological distance from unethical behavior can be considered a form of moral disengagement, which can also be considered in katagelasticism when the psychological distance shapes the benign (instead of harmful) character of a specific violation. Similar findings were reported by Hardy and their collaborators [61], who explored social dominance orientation and the circle of moral regard as forms of psychological distance concerning moral identity and parental socialization. Furthermore, katagelasticists usually express low shame and guilt-proneness and are relatively unconcerned with what happens around them [62]. Thus, a potentially interesting future research direction might be related to an ecological perspective that would explore (a) the mediating role of psychological distance on the relationship between katagelasticism and moral disengagement; (b) katagelasticism as a form of moral disengagement. Furthermore, future studies might also benefit from developing a new scale for measuring katagelasticism as a form of moral disengagement, using the currently identified links with compassion and intolerance of uncertainty.

Intolerance of uncertainty partially mediated the link between compassion and moral disengagement, and this specific result aligns with previous studies that underline similar connections. For example, intolerance of uncertainty was previously significantly associated with moral disengagement [36] and various dark traits, which are further connected to katagelasticism and maladaptive humor styles general [45]. Also, Kuiper et al. [45] suggested that high levels of intolerance of uncertainty led to increased worry and maladaptive humor, which is further connected to moral disengagement, as previous studies suggested [19]. Thus, the relationship between katagelasticism (and maladaptive forms of humor) and uncertainty intolerance seems bidirectional. More specifically, people might use maladaptive humor to cope with elevated stress levels caused by intolerance of uncertainty, which could also lead to moral disengagement. Nevertheless, these potential research directions need further investigation.

On the other hand, compassion was significantly and negatively linked to both katagelasticism and moral disengagement. People who take joy in hurting others using their humor have intuitively lower levels of compassion, and, subsequently, they engage easier in moral disengagement mechanisms that would further ease their potentially uncomfortable state of mind. The moral value of compassion is undeniable, given its role as a source of moral motivation [63]. Thus, it is imperative to emphasize compassion as a desirable trait as early as possible, i.e., starting from kindergarten ages, given its significant role in building moral identity, moral character, and one’s general well-being [64–66]. Furthermore, since humor can comprise both collaborative and competitive motives (despite the verbal contents implied [67], an interesting future research direction might be related to the evolutionary perspective on compassion and the related social dynamics—including humor [68, 69].

Several limitations need to be addressed for the present research. First, all the instruments were self-reported, which might have raised some issues regarding desirability. Future studies might benefit from using alternative measures (e.g., experimental) that would also allow the investigation of causal relationships between the variables. However, in the current circumstances (of the present study), no such causal links could be assessed. Second, the study’s sample was convenient; thus, it cannot be regarded as representative of the larger population [70]. Future studies might address this issue by exploring the links between the proposed variables using different sampling techniques since convenient sampling might lower the study's external validity [71]. Finally, other factors that might have interfered with moral disengagement tendencies were not accounted for, such as empathy, moral identity, reflective moral attentiveness, Machiavellianism, cynicism, and external locus of control [39–42], or the Big Five domains and aspects differently contribute to moral disengagement [72].

## Practical implications and concluding remarks

Overall, I found that individuals who scored low on compassion and high on their intolerance of uncertainty were more prone to dissociate morally and to take pleasure in laughing at the expense of others. The present results shed more light on the complex relational ties between the pleasure derived from laughing at others and the cognitive restructuring that leads to unethical behavior. The practical implications derived from these findings are two-folded. First, these findings highlight the need to generally emphasize that the activation of moral disengagement, further leading to antisocial behavior and adverse psychological effects [73], facilitates the harmful uses of humor.

Furthermore, the current findings highlight the practical need to examine the ethics of humor in a broader context, involving larger age groups and extended morality-related variables (e.g., moral identity, moral relativism), especially due to the significant detrimental effects of moral disengagement. One possible research direction could be using katagelasticism in different cyberbullying contexts and roles (i.e., perpetrators, victims, or passive bystanders). For example, cyber-perpetrators high in katagelasticism might use technology to create denigrating websites or alter one’s imagine in a negative way (to make it *funny)*, as they often justify their unethical behaviors by saying it was *fun* (“It was just a joke!”) [74]. Furthermore, cyber-aggressors who use humor to hurt others usually consider their aggressive acts as ways to “make fun” by using harmful jokes or comments [75, 76].

## Conclusion

To conclude, the present study underlined important connections between the joy of laughing at other people, i.e., katagelasticism, compassion, and the mediating and moderating roles of moral disengagement and intolerance of uncertainty. Though future investigations are needed to clarify the various forms of moral disengagement in relation to humor, the current results highlighted the need for compassion towards others and efficient anxiety management within uncertainty contexts to lower the detrimental effects of disparagement humor and moral disengagement.

## Data Availability

The datasets used and/or analyzed during the current study are available from the corresponding author upon reasonable request.
